# Synergistic Strengthening and Toughening of 3D-Printed Bioinspired Alumina Composites with a Multi-Scale Bouligand Structure

**DOI:** 10.3390/biomimetics11040252

**Published:** 2026-04-06

**Authors:** Zhaozhi Wang, Dongxu Duan, Lei Yang, Xu Bai, Zhibin Jiao, Chenliang Wu, Jing Zhao, Zhihui Zhang

**Affiliations:** 1 School of Mechanical Engineering, Shenyang University of Technology, Shenyang 110870, China; ddx@smail.sut.edu.cn (D.D.); 13722957762@smail.sut.edu.cn (L.Y.); baix@sut.edu.cn (X.B.); jiaozhibin@sut.edu.cn (Z.J.); 2 School of Materials Science and Engineering, Shenyang University of Technology, Shenyang 110870, China; mfclwu@sut.edu.cn; 3 Key Laboratory of Bionic Engineering (Ministry of Education), Jilin University, Changchun 130022, China; zhzh@jlu.edu.cn; 4 Institute of Structured and Architected Materials, Liaoning Academy of Materials, Shenyang 110167, China

**Keywords:** direct ink writing, Bouligand structure, fracture toughness, multiscale reinforcement

## Abstract

Inspired by the Bouligand helicoidal architecture of the dactyl club of the peacock mantis shrimp, this study employed direct ink writing (DIW) 3D printing to construct a three-level synergistic toughening system composed of nano-SiO_2_, microscale flake alumina, and a macroscale helicoidal structure. The effects of nano-SiO_2_ content, Bouligand helix angle, and flake alumina content on the flexural strength and fracture toughness of the composite ceramics were systematically investigated. The results showed that the optimal nano-SiO_2_ addition was 7 wt%, yielding a fracture toughness of 1.03 MPa·m^1/2^, which was 13% higher than that of pure alumina. The introduced intergranular glassy phase transformed the rigid grain-boundary bonding into a moderately strong gradient interface, resulting in higher fracture toughness for all SiO_2_-containing samples than for pure alumina. The Bouligand structure further increased the fracture toughness to a maximum of 1.45 MPa·m^1/2^ at a helix angle of 10°, representing a 39% improvement over the 0° sample. When microscale flake alumina was incorporated into the optimal matrix containing 7 wt% SiO_2_, the best overall mechanical performance was achieved at a flake alumina content of 5 wt%, where the flakes directly dissipated fracture energy through pull-out, fracture, and bridging mechanisms. The synergistic effect of the three structural levels was most pronounced at a helix angle of 20°, at which the sample containing 5 wt% flake alumina achieved a fracture toughness of 2.07 MPa·m^1/2^ with almost no loss in flexural strength, corresponding to a 113% improvement over the sample without flake alumina. These results demonstrate that three-level synergy can be achieved through nanoscale interfacial optimization, microscale energy dissipation by reinforcing phases, and macroscale crack deflection induced by the helicoidal structure, thereby providing important theoretical and experimental support for the multiscale design of high-performance bioinspired ceramic materials.

## 1. Introduction

Alumina ceramics are widely used in aerospace thermal protection components, biomedical implants, and wear-resistant structural parts because of their high hardness, excellent high-temperature resistance, chemical stability, and wear resistance [1,2,3,4]. However, the bonding in alumina ceramics is dominated by covalent and ionic bonds with strong directionality, which restricts dislocation motion under external loading and leads to intrinsic brittleness and poor fracture resistance. As a result, alumina ceramics are prone to catastrophic failure under complex loading conditions, which severely limits their broader application as advanced structural materials. Conventional ceramic forming methods, including dry pressing, plastic forming, and slip casting, together with subsequent drying, debinding, and sintering processes, generally rely on molds [5]. However, owing to the high hardness and brittleness of ceramics, precision machining processes such as grinding, polishing, and cutting remain inherently challenging, often resulting in crack formation, complex processing routes, limited adaptability to components with complex geometries, severe tool wear, and high manufacturing costs [6,7]. Therefore, enhancing the strength and toughness of alumina ceramics without compromising their intrinsic advantages has become a major research focus.

**Figure 1:**
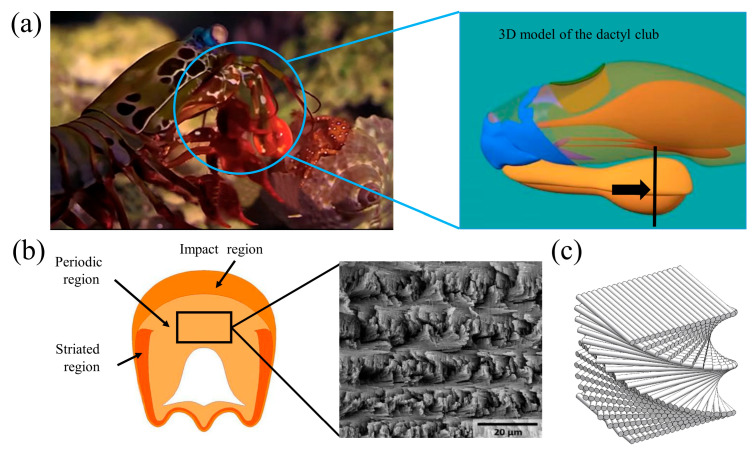
The dactyl club of the peacock mantis shrimp: (**a**) Macroscopic morphology of the dactyl club. (**b**) Microscopic morphology of periodic region. (**c**) Schematic illustration of the Bouligand structure [12].

In recent years, biomimetics has provided a new theoretical framework for the design and fabrication of high-performance ceramic materials [8]. Many natural biological materials offer excellent models for the strengthening and toughening of synthetic materials [9,10,11]. For example, the highly mineralized dactyl club of the peacock mantis shrimp can withstand extreme impact loads without fracturing (Figure 1) [12,13]. From the outer surface inward, the dactyl club consists of three distinct regions, namely the impact region, the periodic region, and the striated region. Specifically, the outermost layer of the impact region is a highly mineralized surface composed of hydroxyapatite nanoparticles, which dissipates impact energy through particle fracture and interparticle sliding [14,15]. Beneath this layer lies a periodic region characterized by fibers arranged in a helicoidal Bouligand structure, which provides exceptional impact resistance [16]. As a crack propagates across interfaces with gradually changing orientations, it is forced to deflect and twist, thereby significantly extending the crack path and dissipating a large amount of fracture energy [17]. Inspired by this mechanism, researchers have successfully introduced the Bouligand structure into layered composite materials and demonstrated its effectiveness in improving fracture toughness [18,19,20,21,22]. Yang et al. [23,24] investigated the fracture behavior of natural Bouligand structures under single-edge notch (SEN) tensile loading. The results indicated that the helicoidal fiber arrangement plays a critical role in the fracture behavior of the Bouligand structure and can effectively filter impact energy. Liu et al. [25] studied the impact resistance of 3D-printed concrete reinforced with steel-fiber-based Bouligand structures. The results showed that concrete with ordered fiber arrangements outperformed randomly distributed fibers, with the ordered fibers bridging cracks under impact loading. Overall, the integration of Bouligand structural design with microscale interfacial engineering may provide an effective strategy for the fabrication of high-performance protective materials.

In addition, the concept of multiscale synergistic reinforcement has attracted increasing attention. The addition of minor alloying elements can alter phase evolution and refine microstructural features, thereby enhancing the mechanical performance of high-entropy alloys [26]. Furthermore, the formation of periodic interfacial layered structures can suppress crack propagation and hinder elemental diffusion, resulting in improved corrosion resistance under aggressive service conditions [27,28]. Bai et al. [29] employed Laser Additive Manufacturing to fabricate metal matrix composites by mixing aluminum alloy powders with silicon carbide (SiC) or boron carbide (B_4_C) powders. They systematically investigated the density, microstructure, and mechanical properties of the materials under different SiC/Al mixing ratios. The study optimized processing parameters such as laser power, scanning speed, and scanning path to achieve the highest densification and mechanical performance. In the research on polymer composites, Ulkir et al. [30] found that in FDM-fabricated polymer composites, material type, infill pattern, and printing direction significantly influence the tensile and flexural strength of the material. Multi-scale synergistic effects are evident in the interactions between different materials, particularly in composite structures, where fiber reinforcement effectively distributes stress and enhances crack resistance. In particulate and ceramic-based systems, the optimization of pore structure, particle packing, and interfacial bonding has also been shown to play a critical role in improving densification, sealing performance, and mechanical reliability [31]. Liu et al. [32] found that by combining 3D printing technology with polymer infiltration into hierarchical porous ceramic lattices, a multi-scale mechanical energy absorption mechanism can be effectively achieved. This structure enhances the permeability of epoxy resin within micro- and nanoscale pores, thereby improving the energy absorption capacity of the composite material. By designing ceramic structures with three levels of porosity, synergistic effects can be achieved at the macro, micro, and sub-micro scales, significantly enhancing the compressive and flexural strength of the material. Nanoscale reinforcing phases, such as nano-SiO_2_, can act as sintering aids by filling grain boundaries and refining grains, and can also react with the Al_2_O_3_ matrix at high temperatures to form mullite, thereby strengthening grain-boundary bonding [33,34]. Meanwhile, microscale flake reinforcing phases, such as flake alumina, can mimic the brick-and-mortar architecture of nacre and effectively dissipate fracture energy through crack deflection, flake bridging, and pull-out [35,36,37,38]. Introducing these two reinforcing phases, which differ in scale and provide complementary functions, into the Al_2_O_3_ matrix is therefore expected to achieve synergistic enhancement of both strength and toughness.

Existing studies have independently confirmed the positive effects of the Bouligand structure and reinforcing phases such as SiO_2_ and flake Al_2_O_3_ on the mechanical properties of ceramic materials. However, most of these studies relied on conventional dry pressing or slip-casting processes, which make it difficult to precisely construct complex macroscopic architectures [39,40]. Three-dimensional printing provides an effective approach for fabricating composite specimens with complex structures. Compared with other ceramic 3D-printing technologies [41,42], the main advantage of DIW lies in the fact that material deposition occurs at room temperature, thereby avoiding thermal effects and laser-induced stress waves during the printing process [43]. DIW 3D printing offers new opportunities for the controlled fabrication of complex bioinspired structures [44,45]. However, systematic studies on DIW-fabricated Bouligand-structured Al_2_O_3_ matrix composites, particularly those examining the synergistic effects of nano-SiO_2_ content, flake Al_2_O_3_ content, and helix angle on mechanical performance, remain scarce.

Although previous studies have explored individual toughening mechanisms, no systematic report has yet addressed their synergistic multiscale interactions in 3D-printed ceramic composites. In addition, previous research has provided only limited quantitative analysis of the role of SiO_2_. In this work, the synergistic effects of three key parameters on the mechanical properties of composite ceramics were systematically investigated over a wide range, including nano-SiO_2_ content (0–11 wt%), Bouligand helix angle (0–20°), and flake alumina content (0–7 wt%). Bioinspired ceramic composites were fabricated by DIW 3D printing. The effects of the selected parameters on the mechanical properties of the composite ceramics were evaluated through mechanical testing, and the synergistic strengthening and toughening mechanisms associated with these three factors were further elucidated through microstructural characterization, including SEM and XRD analyses.

## 2. Materials and Methods

### 2.1. Fabrication of Bioinspired Composites

Matrix alumina powder (alpha-Al_2_O_3_, purity > 99.9%, d_50_ = 1 um, Anhui Estone Material Technology Co., Ltd., Hefei, China), nanoscale silica (SiO_2_, purity > 99.9%, d_50_ = 100 nm, Shanghai Zhongye New Materials Co., Ltd., Shanghai, China), and microscale flake alumina (purity > 99.9%, d_50_ = 5 um, the aspect ratio is 10:1, Sanmenxia Xinfeng Abrasives Co., Ltd., Sanmenxia, China) were dried in a vacuum oven at 100 °C for 48 h to ensure accurate weighing in subsequent steps. Organic solvents were introduced into distilled water to prepare the ink vehicle, including 1 wt% sodium hexametaphosphate (Tianjin Hengxing Chemical Preparation Co., Ltd., Tianjin, China) as a dispersant, 3 wt% polyvinylpyrrolidone (Hefei BASF Bio-technology Co., Ltd., Hefei, China) as a binder, 1 wt% polyethylene glycol (Shanghai Aladdin Biochemical Technology Co., Ltd., Shanghai, China) as a plasticizer, and 0.5 wt% sodium alginate (Shanghai RHAWN Chemical Technology Co., Ltd., Shanghai, China) as a thickener. The mixture was stirred in a planetary vacuum degassing mixer at 600 r/min for 10 min to obtain a homogeneous suspension. Thereafter, the 1 um alumina particles and flake alumina were gradually added in several increments, and each addition was followed by stirring at 800 r/min for 10 min. After mixing, an alumina ceramic slurry with a solid loading of 71 wt% was obtained. The slurry was then placed in a vacuum drying oven for 1 h to remove entrapped air bubbles.

Based on the Bouligand structure of the peacock mantis shrimp dactyl club, a gradient bioinspired strengthening and toughening model was established [46]. Considering that the deflection angle of Bouligand structures in natural materials ranges from 0° to 90°, and taking into account the differences between natural materials and ceramic composites, three deflection angles—10°, 15°, and 20°—were selected for the preparation of bioinspired ceramic samples, with 0° used as the reference. The biomimetic model was designed using 3D modeling software and then imported into the slicing software of the 3D printer. Printing was subsequently performed using a direct ink writing printer. The printing parameters were as follows: nozzle diameter, 0.6 mm; extrusion pressure, 0.1 MPa; nozzle speed, 10 mm/s; layer height, 0.5 mm; and total number of layers, 18, giving a final green-body height of 9 mm. In general, the layer height is maintained at 0.7–0.9 times the nozzle diameter. An appropriate layer height improves interlayer bonding and significantly affects the morphology and mechanical properties of the printed ceramics [47].

**Figure 2:**
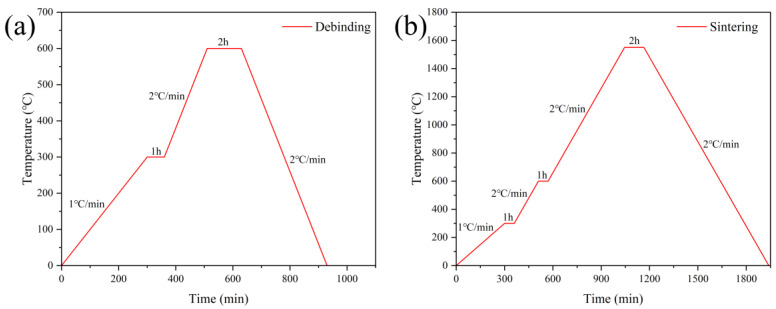
Heat-treatment curves of alumina ceramics: (**a**) debinding; (**b**) sintering.

**Figure 3:**
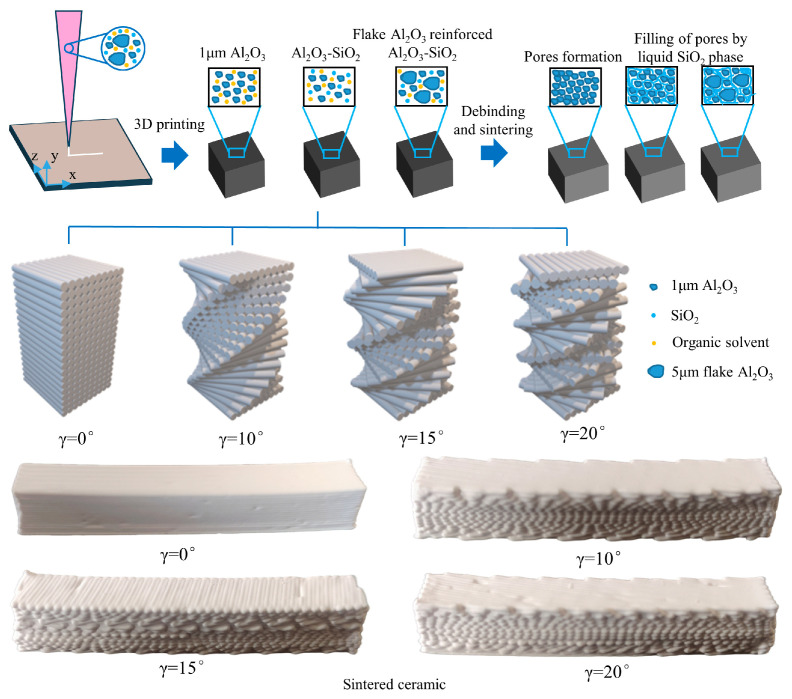
Process flow diagram for the fabrication of 3D-printed bioinspired ceramics.

The heat-treatment process used in this study was as follows. The as-printed green bodies were stored at room temperature for 24 h to allow evaporation of free water, thereby ensuring sufficient drying and reducing the likelihood of deformation during sintering. Debinding was then carried out in a debinding furnace under an air atmosphere, following the profile shown in Figure 2a. The temperature was increased from room temperature to 300 °C at 1 °C/min and held for 1 h, then raised from 300 °C to 600 °C at 2 °C/min and held for 2 h, after which the furnace was allowed to cool naturally to room temperature. After debinding, high-temperature sintering was performed in a sintering furnace under an air atmosphere according to the profile shown in Figure 2b. The heating procedure below 600 °C was identical to that used during debinding. After a 2 h hold at 600 °C, the temperature was increased to 1550 °C at 2 °C/min, held for 2 h, and then naturally cooled to room temperature. Figure 3 shows process flow diagram for the fabrication of 3D-printed bioinspired ceramics.

### 2.2. Mechanical Testing and Characterization

In this study, flexural strength and fracture toughness were measured by three-point bending using a universal testing machine (model UTM6000, Shenzhen Suns Technology Stock Co., Ltd., Shenzhen, China). The flexural strength test was conducted to evaluate the maximum load-bearing capacity of the material under bending stress, specifically the maximum stress that the material can withstand before fracture. This test primarily reflects the mechanical response of the material within the elastic deformation stage and is used to determine its fracture strength under bending conditions. To accurately reflect the true ultimate load-bearing capacity of the material test specimen, the loading rate was set to 0.5 mm/s. In contrast, the fracture toughness test was performed to assess the resistance of the material to crack propagation in the presence of a pre-existing crack. This test focuses on the energy absorption capability of the material, particularly the energy dissipation behavior during crack growth. Compared with the flexural strength test, a lower loading rate is required to more accurately capture the energy evolution and crack propagation process. Therefore, the loading rate for the fracture toughness test was set to 0.05 mm/s. Before sintering, the specimens for flexural strength testing measured 50 mm × 8 mm × 9 mm, and the linear shrinkage after sintering was approximately 5%. The support span was 40 mm. Each condition was tested three times, and the average value was taken as the final result. The flexural strength was calculated according to Equation (1).

(1) $$\sigma =\frac{3FL}{2bh^{2}}$$

In this equation, σ is the flexural strength, F is the break force in the test, L is the outer(support) span, b is the specimen width, and h is the specimen height.

Prior to fracture toughness testing, a pre-crack with a notch width of 0.2 mm and a depth of 2.5 mm was introduced at the center of the bottom surface of each specimen using a diamond blade. Before sintering, the specimens for fracture toughness testing measured 30 mm × 8 mm × 9 mm, with a linear shrinkage of approximately 5% after sintering. The support span was 24 mm. Each condition was tested three times, and the average value was taken as the final result. The fracture toughness was calculated according to Equation (2), where the stress-intensity factor coefficient, *Y*, was determined according to Equation (3).

(2) $$K_{IC}=Y\frac{3FL}{2bh^{2}}\sqrt{a}$$

In this equation, $K_{IC}$ is the fracture toughness, F is the maximum force, L is the outer(support) span, b is the side-to-side dimension of the test specimen perpendicular to the crack length, h is the top-to-bottom dimension of the test specimen parallel to the crack length, a is the crack depth, and Y is the stress intensity factor coefficient, determined by the following equation:

(3) $$Y=1.93-3.07\frac{a}{h}+14.53{\left(\frac{a}{h}\right)}^{2}-25.11{\left(\frac{a}{h}\right)}^{3}+25.8{\left(\frac{a}{h}\right)}^{4}$$

The fracture morphology of the specimens after mechanical testing was examined using a field emission scanning electron microscope (ZEISS GeminiSEM 300, Jena, Germany). Prior to observation, the specimens were cut into dimensions of 1 mm × 1 mm × 1 mm and subsequently sputter-coated with gold to render them electrically conductive. Qualitative and quantitative analyses of the material composition and elemental percentages were conducted using X-ray diffraction (XRD) on an X-ray diffractometer (SmartLab SE, Tokyo, Japan), based on the diffraction of X-rays by the crystal lattice of the material. The operating parameters of the XRD instrument were set as follows: scanning range from 10° to 90°, scanning speed of 2°/min, and step size of 0.02°.

## 3. Results and Discussion

### 3.1. The Influence Mechanism of Nano-Silica Content on the Mechanical Properties of 3D-Printed Alumina Ceramics

**Figure 4:**
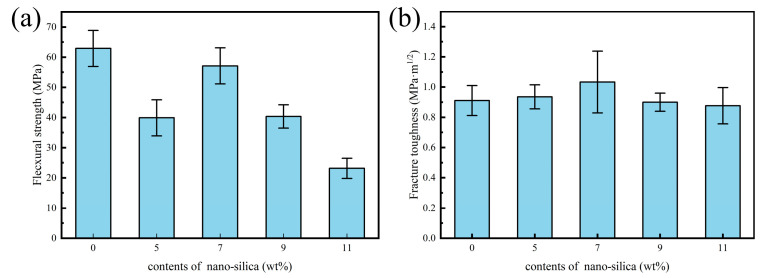
Mechanical properties of ceramics reinforced with different silica contents: (**a**) flexural strength; (**b**) fracture toughness.

Figure 4 compares the flexural strength and fracture toughness of ceramic samples containing different amounts of nano-SiO_2_. With increasing nano-SiO_2_ addition, the flexural strength exhibited a decrease-increase-decrease trend. In contrast, the fracture toughness first increased and then decreased, while all SiO_2_-containing samples exhibited higher toughness than pure alumina.

**Figure 5:**
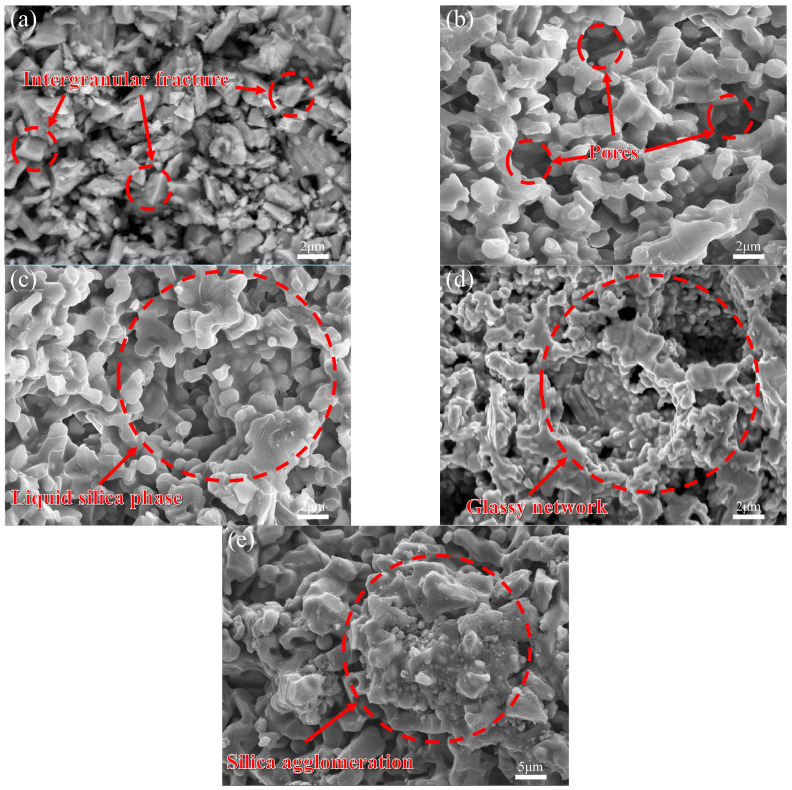
Fracture-surface morphologies of Al_2_O_3_ ceramics with different nano-SiO_2_ contents: (**a**) 0 wt%. (**b**) 5 wt%. (**c**) 7 wt%. (**d**) 9 wt%. (**e**) 11 wt%.

Figure 5 shows SEM images of the fracture surfaces of the samples. As shown in Figure 5a, the grain boundaries are clearly visible. Without a nanoscale reinforcing phase, cracks propagated mainly along the boundaries between alumina grains, and the fracture mode was predominantly intergranular. As shown in Figure 5b, after the addition of 5 wt% nano-SiO_2_, a liquid SiO_2_ phase began to form in the sample. However, the reduction in flexural strength can be attributed mainly to two factors. First, 5 wt% SiO_2_ was insufficient to adequately fill the pores and produce a dense ceramic structure. Second, most of the SiO_2_ formed a discontinuous thin glassy layer at the grain boundaries. Because this glassy phase is weaker than the alumina grains, the sample became more susceptible to intergranular fracture. Nevertheless, the slightly higher fracture toughness compared with pure alumina indicates that the glassy SiO_2_ had already begun to contribute to crack buffering and deflection.

**Figure 6:**
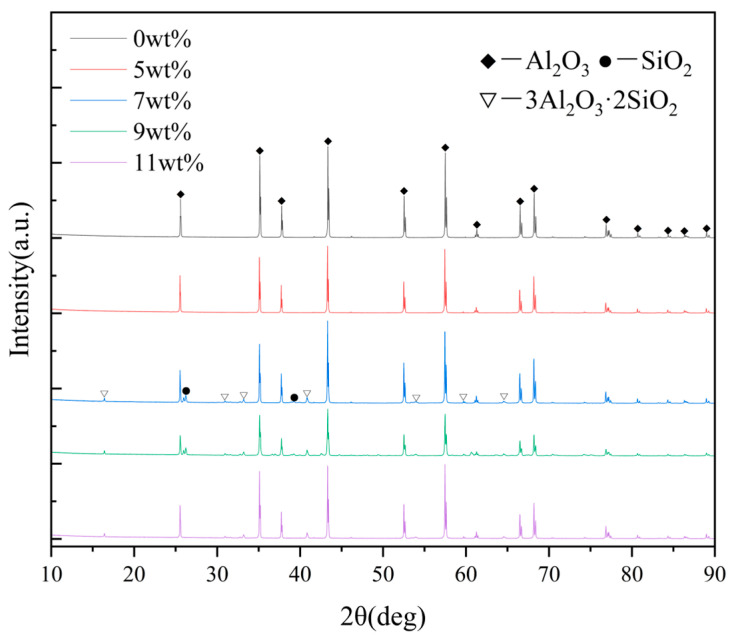
XRD patterns of nano-SiO_2_-reinforced ceramic composites.

When the SiO_2_ content increased to 7 wt%, more liquid SiO_2_ filled the pores of the sample. At the same time, SiO_2_ began to react with Al_2_O_3_ to form mullite (3Al_2_O_3_·2SiO_2_), as confirmed by the XRD results shown in Figure 6. No detectable nano-SiO_2_ or mullite phases were observed in the ceramic containing 5 wt% nano-silica, most likely because the amount of silica added was below the detection limit of the instrument. Mullite possesses high strength and toughness and bonds well with the alumina matrix, thereby producing an in situ reinforcing effect [34,48]. Consequently, the fracture mode changed from intergranular fracture to transgranular fracture, and the flexural strength recovered. At this composition, the amount and distribution of the glass phase reached a favorable balance. The glass phase improved toughness through crack deflection and local plastic deformation, while the presence of mullite maintained sufficient grain-boundary strength. Owing to the synergistic action of these mechanisms, the ceramic containing 7 wt% nano-silica exhibited the best combination of flexural strength and fracture toughness among all SiO_2_-containing samples.

However, when the silica content was further increased to 9 wt% and 11 wt%, the flexural strength decreased again with increasing silica content. This behavior is mainly attributed to the formation of a continuous glassy network along the grain boundaries. Such a continuous glass phase provides a low-strength pathway for crack propagation along the boundaries. In addition, excessive glass phase may encapsulate alumina particles and inhibit grain growth. Moreover, at high SiO_2_ contents, nano-SiO_2_ tends to agglomerate more readily, forming larger clusters, as shown in Figure 5d,e. During sintering, these agglomerates may generate local cracks or large pores because of shrinkage mismatch, thereby becoming new fracture origins.

It is noteworthy that all SiO_2_-containing samples exhibited higher fracture toughness than pure alumina. This suggests that even when the excess glass phase reduces strength, the associated toughening mechanisms remain effective. Overall, 7 wt% nano-SiO_2_ was identified as the optimal content for the synergistic improvement of strength and toughness in this study. Subsequent investigations on the effects of Bouligand helix angle and flake alumina content were therefore conducted using this matrix formulation.

### 3.2. Effect of the Helical Angle of the Biomimetic Bouligand Structure on the Mechanical Properties of Composite Ceramics

**Figure 7:**
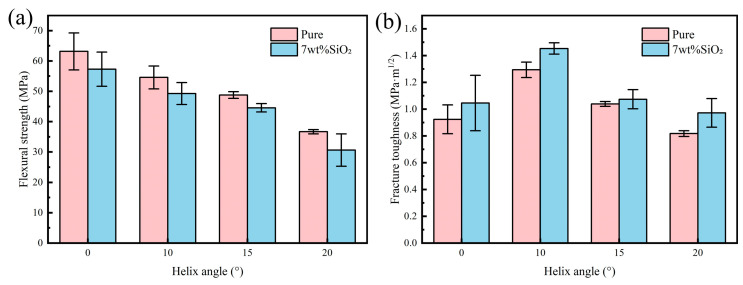
Mechanical properties of pure alumina and nano-silica reinforced ceramic composites: (**a**) flexural strength; (**b**) fracture toughness.

Figure 7 presents the flexural strength and fracture toughness of pure alumina and alumina composite ceramics containing 7 wt% nano-SiO_2_ at four different helix angles: 0°, 10°, 15°, and 20°. The flexural strength results show that both material systems exhibited the same trend: the flexural strength decreased as the helix angle increased. In contrast, the fracture toughness first increased and then decreased, with the 10° sample showing the highest toughness, followed by the 15° sample, whereas the 0° and 20° samples exhibited relatively lower values. These results confirm the effectiveness of the bioinspired Bouligand structure in improving toughness and indicate that its toughening effect is strongly angle-dependent; a larger angle does not necessarily lead to better performance. Except for the pure alumina sample with a 20° Bouligand structure, all other Bouligand-structured samples exhibited higher fracture toughness than the corresponding 0° sample. Together with the earlier observation that the flexural strength of SiO_2_-containing alumina composites was lower than that of pure Al_2_O_3_, these results indicate that either material modification or structural design alone tends to improve toughness at the expense of some strength.

It is noteworthy that the reduction in fracture toughness with increasing helix angle was significantly smaller in the biomimetic samples containing 7 wt% SiO_2_ than in the pure alumina samples. This result indicates that the addition of SiO_2_ partially compensates for the toughness loss induced by increasing helix angle, revealing a synergistic toughening effect with the Bouligand structure. On the one hand, the Bouligand architecture provides a macroscopic geometric framework that promotes crack deflection during fracture. On the other hand, the glass phase introduced by SiO_2_ optimizes the interfacial bonding state and creates an interface of moderate strength. This interface is sufficiently strong to transfer load, yet compliant enough to relieve stress concentration through microscale plastic deformation. As a result, the interfacial failure mode changes from brittle fracture to frictional sliding, which dissipates more energy. Therefore, the fracture toughness of the SiO_2_-containing samples decreased much less sharply with increasing helix angle than that of pure alumina.

**Figure 8:**
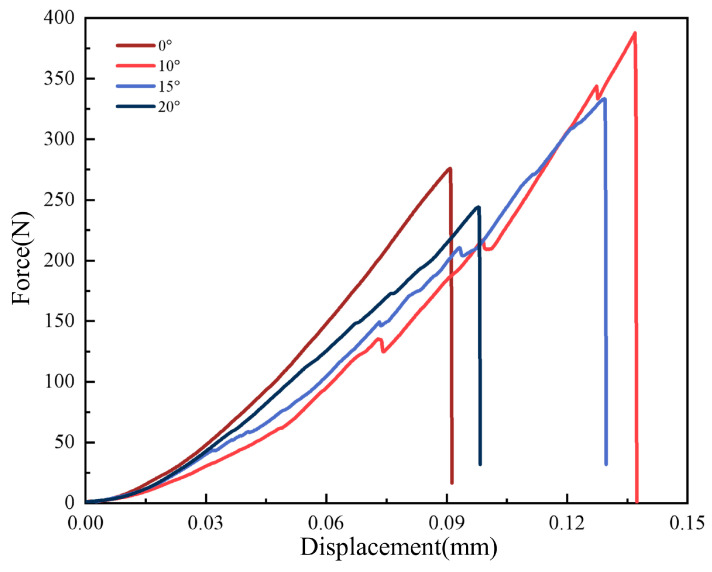
Load–displacement curves obtained from fracture-toughness tests of biomimetic ceramics.

Figure 8 shows the load–displacement curves obtained from fracture toughness tests of Al_2_O_3_-7 wt% SiO_2_ composite ceramics with the four helix angles. The curve of the 0° sample rises smoothly to a peak load and then drops sharply, which is characteristic of brittle fracture and consistent with a nearly straight crack path. By contrast, the curves of the Bouligand-structured samples display pronounced serrated multi-peak features. Each peak corresponds to a resistance event encountered during crack propagation. When a crack reaches an interface with a changed orientation, it is forced to deflect or temporarily arrest, and additional energy must accumulate before propagation can continue. This stepwise process of propagation, arrest, and repropagation represents the microscopic manifestation of the toughening effect of the Bouligand structure. Among the tested samples, the 10° specimen exhibited both the largest fracture displacement and the highest peak load, indicating the longest crack path and the most efficient energy dissipation. The reduced fracture displacement and peak load in the 15° and 20° samples are mainly attributed to the larger helix angle, which increases interfacial shear stress and promotes partial delamination, thereby weakening the overall load-bearing capacity.

The above analysis demonstrates a significant synergistic toughening effect between the Bouligand structure and nano-SiO_2_. The macroscale helicoidal architecture provides a geometric framework for crack deflection and twisting, while the microscale glass phase optimizes the interfacial bonding state and enables this framework to function more effectively. This finding provides an important theoretical basis for the subsequent incorporation of flake alumina to construct a nano-micro-macro multiscale synergistic toughening system.

### 3.3. Effect of Micro-Scale Flake Alumina Content on the Macro-Micro Coupled Strengthening and Toughening Mechanisms of Alumina Composite Ceramics

After identifying Al_2_O_3_-SiO_2_ composite ceramics containing 7 wt% nano-SiO_2_ as the optimal matrix, a microscale flake alumina reinforcing phase was introduced to construct a three-level bioinspired toughening system integrating nanoscale silica, microscale flake alumina, and a macroscale Bouligand structure. The influence of flake alumina content on the mechanical properties of the composite ceramics was then systematically investigated.

**Figure 9:**
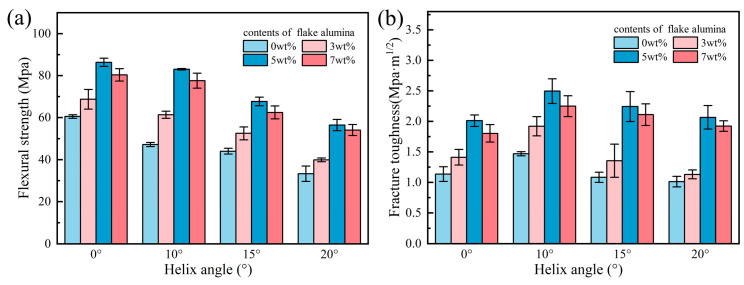
Mechanical properties of Al_2_O_3_-SiO_2_ composite ceramic materials reinforced with different flake alumina contents: (**a**) flexural strength; (**b**) fracture toughness.

**Figure 10:**
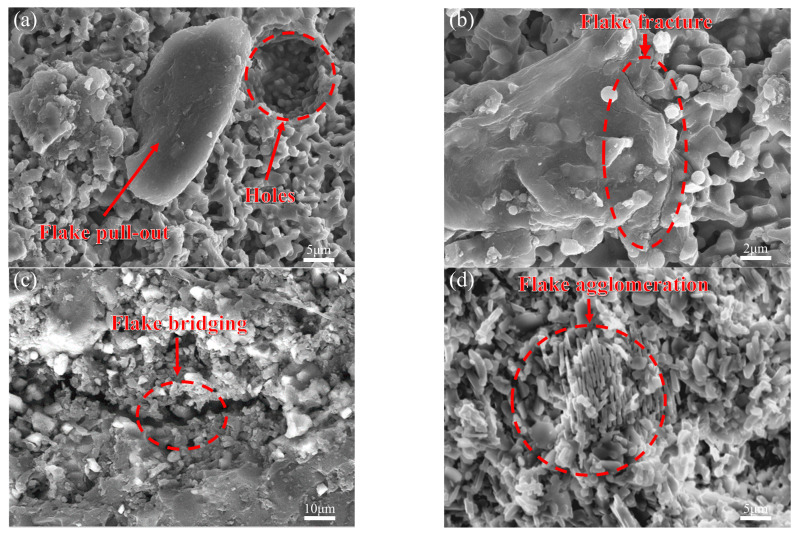
Fracture-surface morphologies of samples containing flake alumina: (**a**) flake pull-out; (**b**) flake fracture; (**c**) flake bridging; (**d**) flake agglomeration.

Figure 9 presents the flexural strength and fracture toughness of 16 groups of samples with four flake alumina contents (0, 3, 5, and 7 wt%) and four Bouligand helix angles (0°, 10°, 15°, and 20°). The addition of flake alumina further improved both the strength and fracture toughness of the ceramic samples. Figure 10 shows SEM images of the fracture surfaces of samples containing flake alumina, clearly revealing four typical behaviors of the flakes during fracture: pull-out, fracture, bridging, and agglomeration.

For a given helix angle, both flexural strength and fracture toughness first increased and then decreased with increasing flake alumina content. The optimal overall mechanical performance was achieved at a flake alumina content of 5 wt%. At 3 wt% and 7 wt%, the properties were slightly lower, but still superior to those of the corresponding samples without flake alumina. This trend was consistent across all four helix angles, indicating that the strengthening and toughening effect of flake alumina is effective over a broad angular range. The microscopic mechanisms can be explained by the fracture surface morphologies shown in Figure 10. Flake alumina dissipates energy mainly through three mechanisms: pull-out (Figure 10a), flake fracture (Figure 10b), and bridging (Figure 10c). At 3 wt%, the amount of flake reinforcement was insufficient to form an effective load-transfer network, resulting in only limited strengthening. At 5 wt%, the flakes were uniformly dispersed throughout the matrix with appropriate interparticle spacing, enabling efficient load transfer through interfacial shear and allowing the three energy-dissipation mechanisms to reach an optimal balance. However, when the content increased to 7 wt%, the excess flakes agglomerated and stacked, preventing them from functioning independently as effective reinforcing units, as shown in Figure 10d. Moreover, microcracks associated with these agglomerates acted as new stress concentrators and preferential sites for crack initiation and propagation, thereby reducing the mechanical properties relative to those of the 5 wt% sample. It is important to note that the effective operation of these toughening mechanisms also depends on the interfacial state optimized by nano-SiO_2_. The SiO_2_-derived glassy phase ensures that the interface between the flakes and the matrix is strong enough to transfer load and permit flake fracture (Figure 10b), while still allowing energy dissipation through pull-out under suitable conditions (Figure 10a). Thus, the interface optimized by nano-SiO_2_ provides the essential foundation for fully realizing the toughening effect of the larger-scale flake alumina.

For a given flake alumina content, the flexural strength still decreased monotonically as the helix angle increased. However, an important observation is that the loss in strength caused by increasing helix angle was significantly smaller in samples containing flake alumina than in those without it. Taking the sample containing 5 wt% flake alumina as an example, the flexural strength losses at helix angles of 10°, 15°, and 20° relative to the 0° sample were 3.81%, 21.6%, and 34.63%, respectively. In contrast, the corresponding losses in the sample without flake alumina were 14.03%, 22.22%, and 46.56%. This phenomenon reveals the reinforcing effect of flake alumina on the Bouligand architecture. Specifically, when the helix angle increases and interlaminar shear stress rises, flakes spanning adjacent layers can resist relative sliding through a bridging effect, thereby enhancing interlaminar shear resistance. This reinforcing effect becomes particularly pronounced at large helix angles. At 20°, the flexural strength of the samples containing 5 wt% and 7 wt% flake alumina increased by 84% and 77%, respectively, compared with that of the corresponding sample without flake alumina. These values approach the flexural strength of the 0° sample without flake alumina. In other words, by introducing an appropriate amount of flake alumina, the toughening benefit of the Bouligand structure can be achieved with almost no loss in strength.

For a given flake alumina content, the fracture toughness also exhibited a rise-and-fall trend with increasing helix angle. The fracture toughness reached its maximum at 10° for all samples and then decreased as the angle increased. This trend is consistent with that observed for composite ceramics without flake alumina in Section 3.2, again indicating that 10° represents the optimal balance point for the present material system. However, the results in this section reveal a more important phenomenon: the toughness increase induced by flake alumina became even more significant at helix angles of 15° and 20°. For example, in the sample containing 5 wt% flake alumina, the fracture toughness at 10° reached 2.50 MPa·m^1/2^, corresponding to a 72% increase over the value of 1.45 MPa·m^1/2^ for the sample without flake alumina. At 15° and 20°, the fracture toughness increased from 1.07 and 0.97 MPa·m^1/2^ to 2.24 and 2.07 MPa·m^1/2^, respectively, corresponding to improvements of 109% and 113%.

The results of this study indicate that the mechanical properties of bioinspired alumina composite ceramics are governed not by a single factor, but by the coupled effects of nano-SiO_2_ content, Bouligand helix angle, and flake alumina content. The nano–micro–macro three-level synergistic toughening architecture enables two coupled crack-propagation modes in ceramic specimens with large-angle helicoidal structures. One mode is interlaminar twisting guided by the macroscopic Bouligand structure, which enhances fracture resistance by forcing cracks to deflect and twist between adjacent layers with progressively changing orientations. Although a larger helix angle strengthens this geometric crack-guiding effect, it also increases interlaminar shear stress and the tendency for local interfacial delamination. Therefore, the toughening efficiency of the Bouligand structure depends not only on its geometric ability to deflect cracks, but also on whether the interface is sufficiently optimized to accommodate the stress redistribution induced by the helicoidal architecture. The other mode is microscale crack deflection induced by the local orientation of flake alumina, which dissipates fracture energy primarily through bridging, pull-out, and flake fracture. However, its toughening effect also depends strongly on the flake content and the surrounding structural environment. At a flake alumina content of 5 wt%, the flakes are more uniformly dispersed and can interact more effectively with propagating cracks. More importantly, under the 15° and 20° conditions, the contribution of flake alumina becomes particularly pronounced because the stronger interlaminar activity provides greater opportunities for flake bridging and frictional energy dissipation. In this context, flake alumina not only directly enhances the fracture resistance of the matrix but also compensates for the adverse effects associated with large-angle helicoidal structures by suppressing interlaminar relative sliding and stabilizing the activated interfaces. The superposition of these two deflection mechanisms gives rise to a complex three-dimensional crack path with both macroscopic helicoidal features and microscopic tortuosity. Meanwhile, the increase in interfacial shear stress at larger angles promotes micro-interfacial separation, which in turn activates the bridging mechanism of the flakes, allowing more fracture energy to be dissipated through interfacial frictional sliding. The interface optimized by nano-SiO_2_ is essential for this behavior. It is neither so strong that the flakes fracture directly, nor so weak that pull-out becomes ineffective. As a result, at 15° and 20°, the increase in fracture toughness exceeds that observed at 10°. The full realization of this mechanism relies on the nano-SiO_2_-modified interface, which provides an appropriate balance between interfacial bonding strength and debonding resistance. Therefore, the excellent toughness exhibited by the composite ceramic containing 7 wt% nano-SiO_2_, 5 wt% flake alumina, and a 20° Bouligand structure should be regarded as a true nano–micro–macro multiscale synergistic toughening effect arising from the combined action of interfacial regulation, helicoidal crack guidance, and flake-mediated energy dissipation. These results demonstrate that the optimal design of bioinspired ceramic composites should be based on the coordinated matching of composition, structure, and reinforcing phases, rather than on the isolated maximization of any single parameter.

## 4. Conclusions

In this work, alumina–matrix composite ceramics inspired by the Bouligand structure of the peacock mantis shrimp were successfully fabricated by DIW 3D printing. A three-level synergistic toughening system consisting of nanoscale SiO_2_, microscale flake alumina, and a macroscale Bouligand structure was established. The effects of compositional parameters and structural design on the mechanical properties were systematically investigated, and the underlying multiscale coupled strengthening and toughening mechanisms were elucidated. Within the material system studied, the introduction of nano-SiO_2_ led to an overall improvement in fracture toughness. The grain-boundary glass phase formed by nano-SiO_2_ transformed the rigid grain-boundary bonding into a moderately strong gradient interface, thereby providing the basis for subsequent toughening mechanisms. The optimal nano-SiO_2_ content was 7 wt%, at which the fracture toughness reached 1.03 MPa·m^1/2^, representing a 13% increase over pure alumina. The Bouligand structure further improved fracture toughness, which reached a maximum of 1.45 MPa·m^1/2^ at a helix angle of 10°, corresponding to a 39% increase over the 0° sample. Meanwhile, the buffering effect associated with SiO_2_ became more evident at 20°, indicating an initial synergy in which toughness was enhanced at a relatively small cost in strength. Based on the optimized matrix containing 7 wt% SiO_2_, the introduction of 5 wt% microscale flake alumina further improved the mechanical performance. The resulting composite achieved a fracture toughness of 2.07 MPa·m^1/2^ with almost no loss in flexural strength, corresponding to a 113% increase over the sample without flake alumina. The flakes dissipated fracture energy directly through pull-out, fracture, and bridging mechanisms. Moreover, the synergistic effect of the three components became more pronounced at larger helix angles. Most importantly, this work demonstrates that a multiscale bioinspired design strategy can effectively overcome the conventional strength–toughness trade-off in ceramics, thereby enabling the simultaneous optimization of damage tolerance and mechanical integrity. This study therefore provides an important theoretical basis and practical design strategy for the development of high-performance bioinspired ceramic materials. This design concept offers a promising pathway for guiding the future development of high-performance structural ceramics with enhanced reliability, adaptability, and multifunctional potential.

## Data Availability

The original contributions presented in this study are included in the article. Further inquiries can be directed to the corresponding author.
